# Feasibility of nodal classification for non‐small cell lung cancer by merging current N categories with the number of involved lymph node stations

**DOI:** 10.1111/1759-7714.13094

**Published:** 2019-06-17

**Authors:** Wei Chen, Chenlei Zhang, Gebang Wang, Zhanwu Yu, Hongxu Liu

**Affiliations:** ^1^ Department of Thoracic Surgery Cancer Hospital of China Medical University, Liaoning Cancer Hospital & Institute Shenyang China

**Keywords:** Lung cancer staging, lymph node classification, lymph node station, N categories, non‐small cell lung cancer

## Abstract

**Introduction:**

The aim of this study was to assess the prognoses of patients with non‐small cell lung cancer (NSCLC) according to the current nodal (N) categories of the tumor, node and metastasis (TNM) classification and the number of involved lymph node stations.

**Methods:**

Five hundred and seventy patients with NSCLC underwent surgery from 1 January 2005 to 31 December 2009 and were analysed retrospectively. Postoperative overall survival was analysed according to two nodal classifications: the current N0, N1, N2 and N3 categories and those based on the number of involved nodal stations: N0, N1a (single N1), N1b (multiple N1), N2a1 (single N2 without N1), N2a2 (single N2 with N1), N2b1 (multiple N2 without N1) and N2b2 (multiple N2 with N1).

**Results:**

Five‐year survival rates were 76.1%, 53.4% and 26.3% for N0, N1 and N2, respectively (*P* < 0.001). When survival was analysed by the number of involved nodal stations, the groups with significant differences were maintained; otherwise, they were merged, and new codes were assigned as follows for exploratory analyses: NA (N0), NB (N1a), NC (N1b, N2a (i.e., N2a1 and N2a2) and N2b1) and ND (N2b2). Five‐year survival rates were 76.1%, 60.0%, 39.1%, and 11.4% for NA, NB, NC and ND, respectively, and there were significant differences among them. This N classification was an independent prognostic factor in multivariate analyses.

**Conclusion:**

Pending prospective and international validation, it is practical to merge the current N categories with the number of involved lymph node stations when evaluating the postoperative prognosis of NSCLC patients.

## Introduction

The eighth edition of the tumor, node and metastasis (TNM) classification for lung cancer, published in 2016,^1^, ^2^, ^3^ is widely used in clinical practice and universally recognized by clinicians. Accurate staging of lung cancer is a very important basis for clinicians to provide appropriate treatment strategies, including surgery, radiotherapy, chemotherapy, and targeted therapy, and assess patient prognosis. Compared with the previous edition, in the eighth edition, there were several significant changes with regard for T and M descriptors and stages.^4^, ^5^, ^6^

The current nodal classification (N) of lung cancer is defined by the anatomical locations of involved lymph nodes: N0 (no regional lymph node metastasis), N1 (metastasis in ipsilateral peribronchial or ipsilateral hilar lymph nodes or intrapulmonary nodes, including involvement by direct extension), N2 (metastasis in ipsilateral mediastinal or subcarinal lymph nodes), and N3 (metastasis in contralateral mediastinal, contralateral hilar, ipsilateral or contralateral scalene, or supraclavicular lymph nodes).^7^ There was no need to modify these categories because they effectively predicted and distinguished the prognosis of patients with involvement of different lymph nodes.^8^ However, this method of lymph node classification simply considers the influence of the location of lymph node involvement and does not analyse the relationships between survival and other conditions related to metastatic lymph nodes.

Although no revision was made to the N component, the International Association for the Study of Lung Cancer (IASLC) explored the prognostic implications of the number of involved nodal stations.^8^ Similarly, the impact of the number of involved nodal zones was analysed for the seventh edition.^9^ Based on pathologic staging, the number of involved nodal stations (nLNS) can be used to subclassify the present N categories: N1a, involvement of a single N1 station; N1b, involvement of multiple N1 stations; N2a1, involvement of a single N2 station without N1; N2a2, involvement of a single N2 with N1; and N2b, involvement of multiple N2 stations. There is a statistically significant degradation in survival as patients move from through stages from N1a to N2b, except for N1b and N2a1, which have similar prognoses.^8^

According to this idea, this study evaluates the clinical feasibility of using a lymph node classification that combines N with nLNS (N‐nLNS).

## Methods

### Clinical data

From 1 January 2005 to 31 December 2009, a total of 1348 patients who underwent lung resection for lung cancer in the Department of Thoracic Surgery, Cancer Hospital of China Medical University, Liaoning Cancer Hospital & Institute (Shenyang, Liaoning province, China) were included in this study. Collected clinical data included age, sex, smoking history (the World Health Organization [WHO] defines never‐smokers as those who either have never smoked or have smoked less than one hundred cigarettes in their lifetime^10^), the location of the lung tumor, the postoperative pathological type, T and N descriptors, the acquisition and metastasis of lymph nodes, survival, the TNM stage and the follow‐up period. All tumors were staged according to the eighth edition of TNM classification for lung cancer.^7^ Pathological diagnoses were performed according to the latest WHO classification.^11^ The Internal Review Board of the Cancer Hospital of China Medical University, Liaoning Cancer Hospital & Institute approved the study. Due to the anonymized nature of the study, informed consent was waived.

### Inclusion and exclusion criteria

#### Inclusion criteria

(i) Tumor resection; (ii) the pathological diagnosis was non‐small cell lung cancer (NSCLC); (iii) the surgical procedure was lobectomy; (iv) complete systematic lymph node dissection (the number of removed lymph node stations in both the N1 and N2 groups, including the subcarinal station, was greater than or equal to three); and (v) a complete record of the clinical data was available.

#### Exclusion criteria

(i) Only nonsurgical treatment, such as radiotherapy and chemotherapy, or preoperative neoadjuvant therapy was performed; (ii) the pathological diagnosis was small cell lung cancer (SCLC); (iii) the surgical procedure was sublobar resection (wedge resection or segmentectomy); (iv) without complete systematic lymph node dissection; (v) clinical information was lost or recorded incompletely; and (vi) failed to complete the follow‐up period.

### Preoperative staging

Clinical staging was performed by imaging examination (e.g., lung computed tomography [CT] and positron emission tomography‐computed tomography [PET‐CT], Emission Computed Tomography [ECT], brain CT or magnetic resonance [MR]) and biopsy (e.g., supraclavicular lymph node biopsy, mediastinoscopy, endoscopic ultrasound‐guided biopsy). Lymph node biopsies were performed in patients with highly suspicious mediastinal N2 or supraclavicular N3 disease based on findings on chest CT, ultrasound and/or PET‐CT.

### Classification of nodal disease

Survival analyses were performed according to two different types of nodal classification: the current N categories, including N0, N1, N2 and N3 (N); and the proposed categories based on the number of involved lymph node stations, including N0, N1a, N1b, N2a1, N2a2 and N2b (nLNS).^8^ In addition, for the purpose of this study, N2b was subclassified into N2b1 (multiple N2 stations involved but without N1) and N2b2 (multiple N2 stations involved with N1).

### Survival and follow‐up

Survival was the primary outcome and was measured from the date of surgery for all patients. Follow‐up was mainly conducted through outpatient reviews, telephone interviews, and other forms. Death from any cause or lost to follow‐up were end‐points, and the last follow‐up time was 7 May 2017.

### Statistical analyses

Postoperative survival was assessed using the Kaplan‐Meier method. The Cox regression model was applied to analyse the clinicopathological factors that influenced overall survival by univariate and multivariate analyses. All tests were two‐sided, and a *P*‐value < 0.05 was considered statistically significant. All analyses were performed using SPSS 22.0 software (SPSS Inc., Chicago, IL, USA).

## Results

### Patients’ characteristics

**Figure 1:**
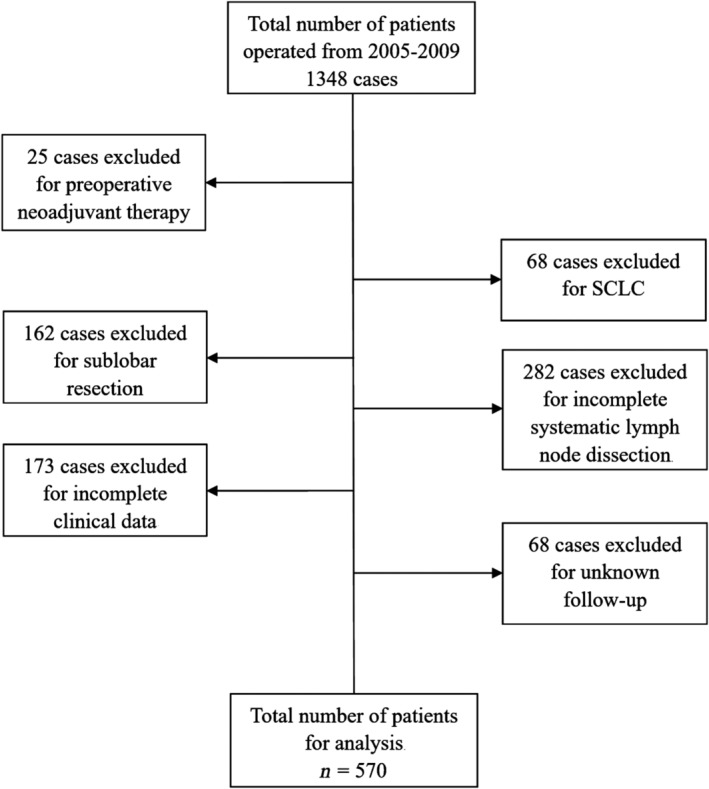
The flow chart of the procedure of patient selection.

After rigorous screening (Fig. 1), a total of 570 cases were available for analysis. Their general clinicopathological characteristics are shown in Table 1. The mean age was 57.8 years old (20–83 years old). Most patients (355 cases, 62.3%) were male, and the male:female ratio was 1.65:1. A smoking history was found in 53.7% of the patients. There was no clear difference in the number of patients whose lesions were located in the right (305 cases, 53.5%) or left (265 cases, 46.5%) lung. The most common postoperative pathological type was adenocarcinoma (317 cases, 55.6%). The majority of tumors were pT1 (224 cases, 39.3%) or pT2 (230 cases, 40.3%). The proportions of N0 (330 cases, 57.9%) were predominant. There was no patient with stage IV disease in our study. The clinicopathological characteristics of different N categories are summarized in Table 2. The distributions according to age, sex, smoking history, the location of the tumor and the pathological type were similar among the N0, N1, and N3 groups. There was only one case with pT4 in the N1 group. There was no stage I disease in the N1 cases, and all N2 cases were classified as stage III. The number of collected lymph node stations was six or more in each case. The number of lymph nodes harvested in each station ranged from 1 to 12 (the mean value was 1.6), and the number of positive lymph nodes ranged from 0 to 9 (the mean value was 0.2).

**Table 1:** Clinicopathological characteristics of 570 patients with completely resected non‐small cell lung cancer

Clinicopathological characteristics	*n* (%)
Age	
<60 years	337 (59.1)
≥60 years	233 (40.9)
Sex	
Male	355 (62.3)
Female	215 (37.7)
Smoking history	
Yes	306 (53.7)
No	264 (46.3)
Location of tumor	
Right lung	305 (53.5)
Left lung	265 (46.5)
Pathological type	
Adenocarcinoma	317 (55.6)
Squamous cell carcinoma	217 (38.1)
Others	36 (6.3)
pT categories	
pT1	224 (39.3)
pT2	230 (40.3)
pT3	82 (14.4)
pT4	34 (6.0)
N categories	
N0	330 (57.9)
N1	88 (15.4)
N2	152 (26.7)
TNM stage	
I	224 (39.3)
II	155 (27.2)
III	191 (33.5)

**Table 2:** Clinicopathological characteristics of N0, N1 and N2 cases

Clinicopathological characteristics	N0 (330 cases)	N1 (88 cases)	N2 (152 cases)
Age			
<60 years	176 (53.5%)	55 (62.5%)	106 (69.7%)
≥60 years	154 (46.7%)	33 (37.5)	46 (30.3%)
Sex			
Male	213 (64.5%)	54 (61.4%)	88 (57.9%)
Female	117 (35.5%)	34 (38.6%)	64 (42.1%)
Smoking history			
Yes	182 (55.2%)	50 (56.8%)	74 (48.7%)
No	148 (44.8%)	38 (43.2%)	78 (51.3%)
Location of tumor			
Right lung	181 (54.8%)	41 (46.6%)	83 (54.6%)
Left lung	149 (45.2%)	47 (53.4%)	69 (45.4%)
Pathological type			
Adenocarcinoma	172 (52.1%)	42 (47.7%)	103 (67.8%)
Squamous cell carcinoma	135 (40.9%)	39 (44.3%)	43 (28.3%)
Others	23 (7.0%)	7 (8.0%)	6 (3.9%)
pT categories			
pT1	155 (47.0%)	26 (29.6%)	43 (28.3%)
pT2	114 (34.5%)	44 (50.0%)	72 (47.4%)
pT3	40 (12.1%)	17 (19.3%)	25 (16.4%)
pT4	21 (6.4%)	1 (1.1%)	12 (7.9%)
TNM stage			
I	224 (67.9%)	0 (0)	(0)
II	85 (25.7%)	70 (79.5%)	(0)
III	21 (6.4%)	18 (20.5%)	152 (100%)

### Prognosis of N categories

**Figure 2:**
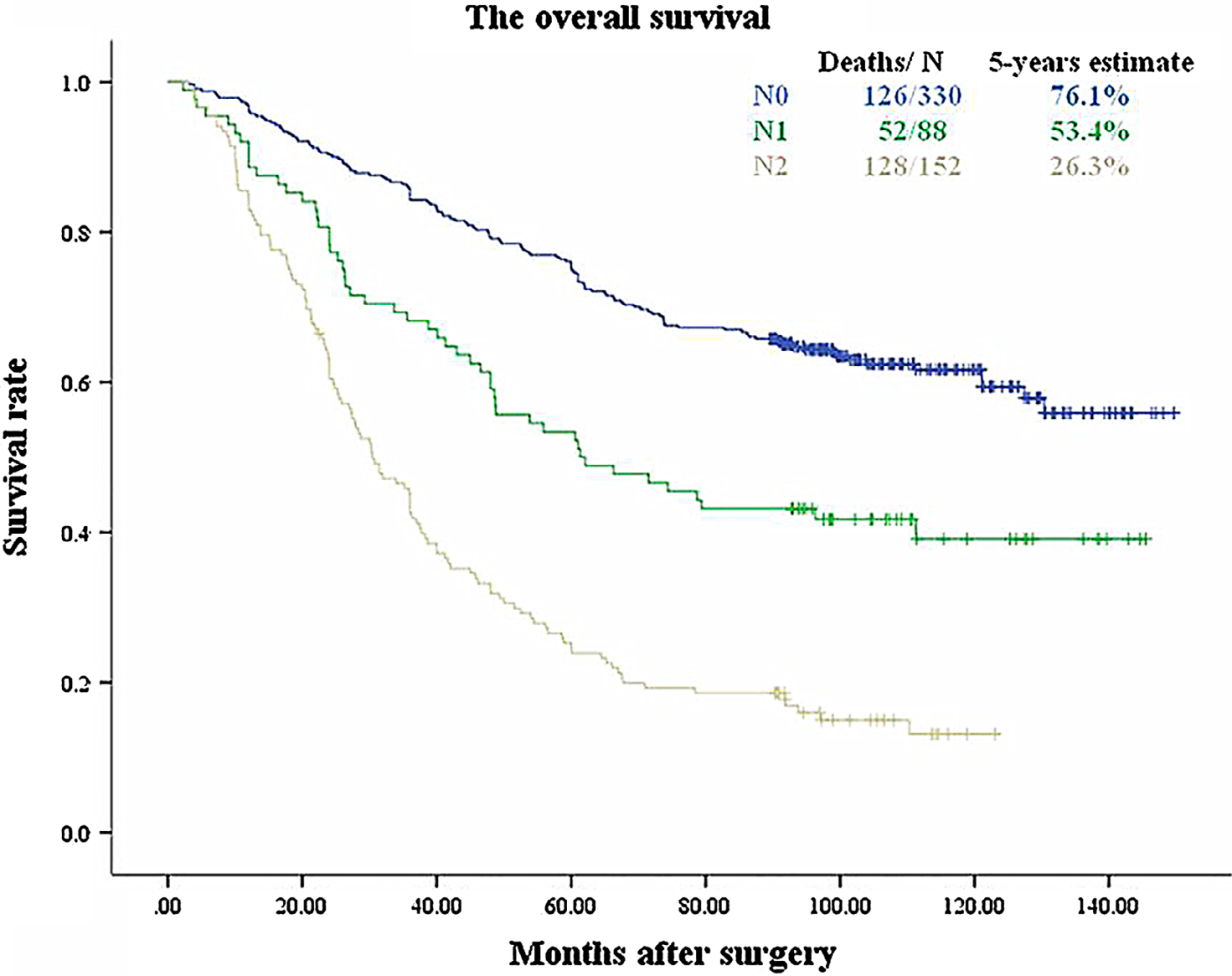
Survival curves for N0, N1 and N2 of N (Log Rank *P* < 0.001). The differences of survival between neighboring groups were statistically significant (*P* values: between N0 and N1, *P* < 0.001; between N1 and N2, *P* < 0.001).

When categorized according to the N categories, the tumors found in this study were divided into N0 (330 cases, 57.9%), N1 (88 cases, 15.4%), and N2 (152 cases, 26.7%), and there were no N3 cases. The five‐year survival rates were 76.1%, 53.4%, and 26.3% for N0, N1 and N2, respectively (between N0 and N1, *P* < 0.001; between N1 and N2, *P* < 0.001) (Fig. 2). In the univariate analyses, the N categories and pT categories were associated with survival. In the multivariate analyses, N categories were independent risk factors that affected patient survival (Table 3).

**Table 3:** Results of univariate and multivariate analyses of N (Cox regression model)

	Univariate analyses			Multivariate analyses		
	HR	95%CI	*P*‐value	HR	95%CI	*P*‐value
N			<0.001			<0.001
N0 versus N1	0.518	0.375–0.716	<0.001	0.537	0.386–0.746	<0.001
N1 versus N2	0.452	0.327–0.626	<0.001	0.486	0.350–0.674	<0.001
Age						
<60 years versus ≥60 years	0.896	0.715–1.124	0.344			
Sex						
Male versus female	1.161	0.918–1.467	0.212			
Smoking history						
Yes versus No	1.221	0.974–1.531	0.084			
Location of tumor						
Right lung versus left lung	0.850	0.680–1.064	0.157			
Pathological type			0.526			
Adenocarcinoma versus squamous cell carcinoma	0.988	0.780–1.251	0.920			
Adenocarcinoma versus others	0.775	0.497–1.209	0.261			
Squamous cell carcinoma versus others	0.785	0.498–1.237	0.297			
pT categories			<0.001			<0.001
pT1 versus pT2	0.566	0.432–0.742	<0.001	0.700	0.532–0.922	0.011
pT2 versus pT3	0.653	0.480–0.889	0.007	0.698	0.513–0.952	0.023
pT3 versus pT4	0.602	0.383–0.946	0.028	0.521	0.329–0.824	0.005

### Combining N with nLNS to generate N‐nLNS categories

**Figure 3:**
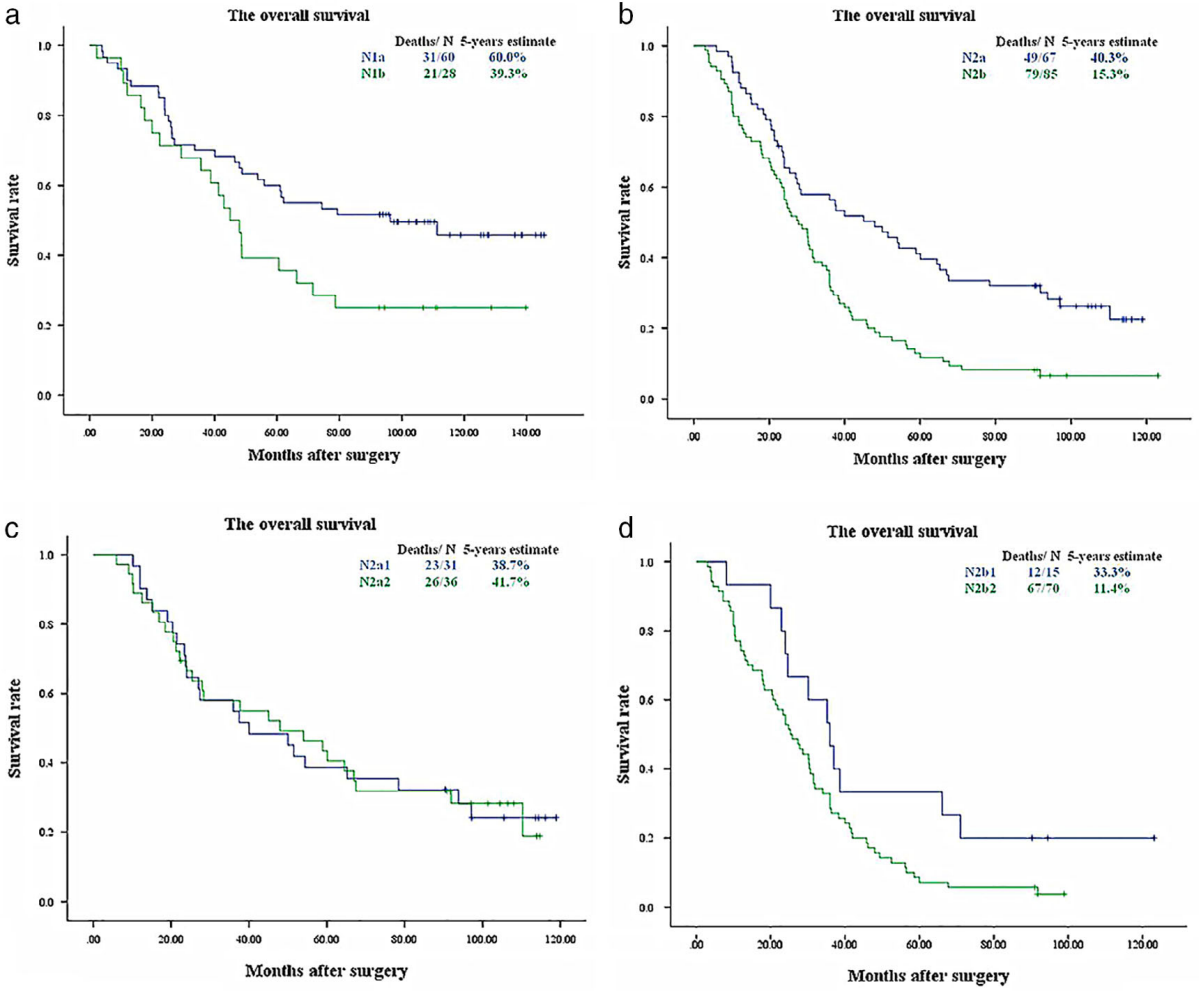
(**a**) Survival curves for N1a and N1b (Log Rank *P* = 0.032). (**b**) Survival curves for N2a and N2b (Log Rank *P* < 0.001). (**c**) Survival curves for N2a1 and N2a2 (Log Rank *P* = 0.997). (**d**) Survival curves for N2b1 and N2b2 (Log Rank *P* = 0.043).

**Figure 4:**
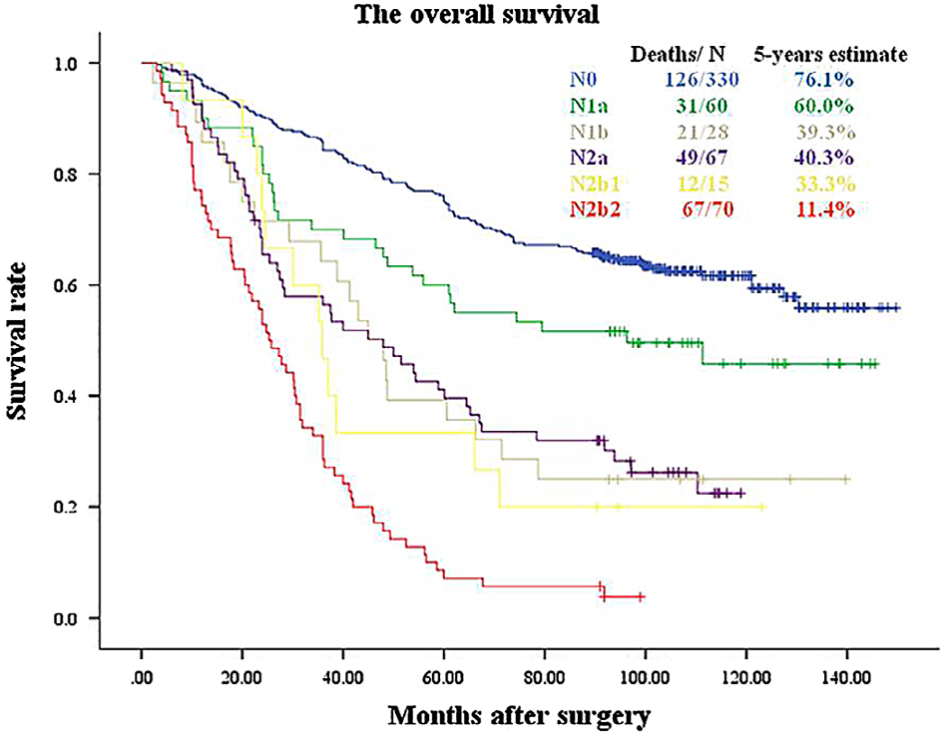
Survival curves for N0, N1a, N1b, N2a, N2b1 and N2b2 (Log Rank *P* < 0.001).

**Figure 5:**
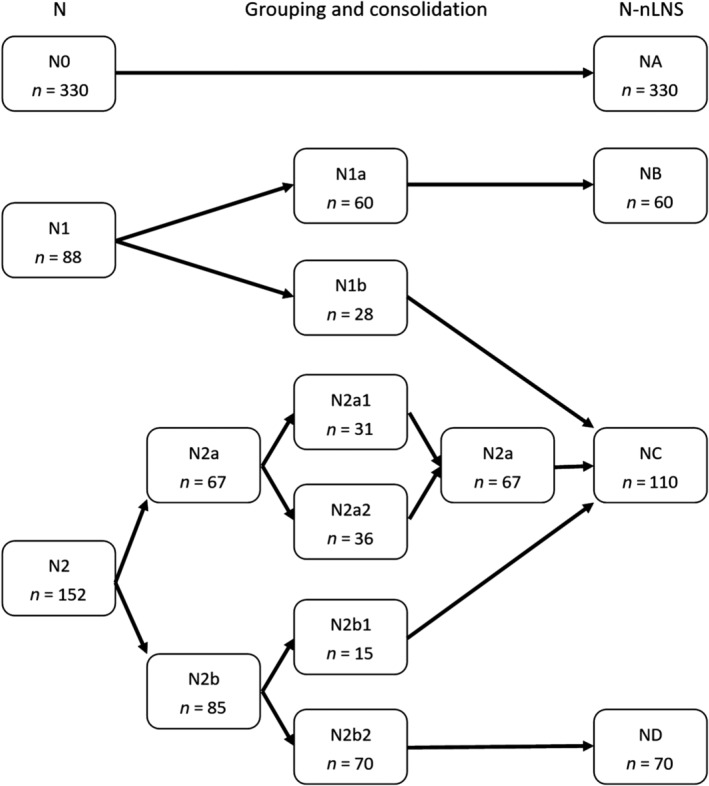
The procedure of grouping and consolidation.

N categories and nLNS were combined, and the prognostic impact of categories on patients with NSCLC was further explored in this study. As shown in Figure 3a, survival times were significantly longer in patients with N1a than in those with N1b (*P* = 0.032). The prognosis after surgery was better in patients with N2a than in those with N2b (*P* < 0.001) (Fig. 3b). Figure 3c shows that there was no statistically significant difference in survival between N2a1 and N2a2 (P = 0.997). However, there was a statistically significant difference in survival between patients with N2b1 and N2b2 tumors (Fig. 3d). Additionally, Figure 4 and Table 4 show that N1b, N2a, and N2b1 tumors (between N1b and N2a, *P* = 0.967; between N1b and N2b1, *P* = 0.559; between N2a and N2b1, *P* = 0.614) could be grouped together into one set. For an exploratory analysis, these nodal categories were coded as NA (original N0), NB (original N1a), NC (original N1b, N2a, and N2b1) and ND (original N2b2) (Fig. 5).

**Table 4:** Paired comparisons of differences in survival rates between N0, N1a, N1b, N2a, N2b1 and N2b2

			*P*‐value				*P*‐value
N0	versus	N1a	0.023	N1b	versus	N2a	0.967
		N1b	<0.001			N2b1	0.559
		N2a	<0.001			N2b2	0.001
		N2b1	<0.001	N2a	versus	N2b1	0.614
		N2b2	<0.001			N2b2	<0.001
N1a	versus	N1b	0.032	N2b1	versus	N2b2	0.043
		N2a	0.009				
		N2b1	0.027				
		N2b2	<0.001				

### Prognosis of N‐nLNS categories

**Figure 6:**
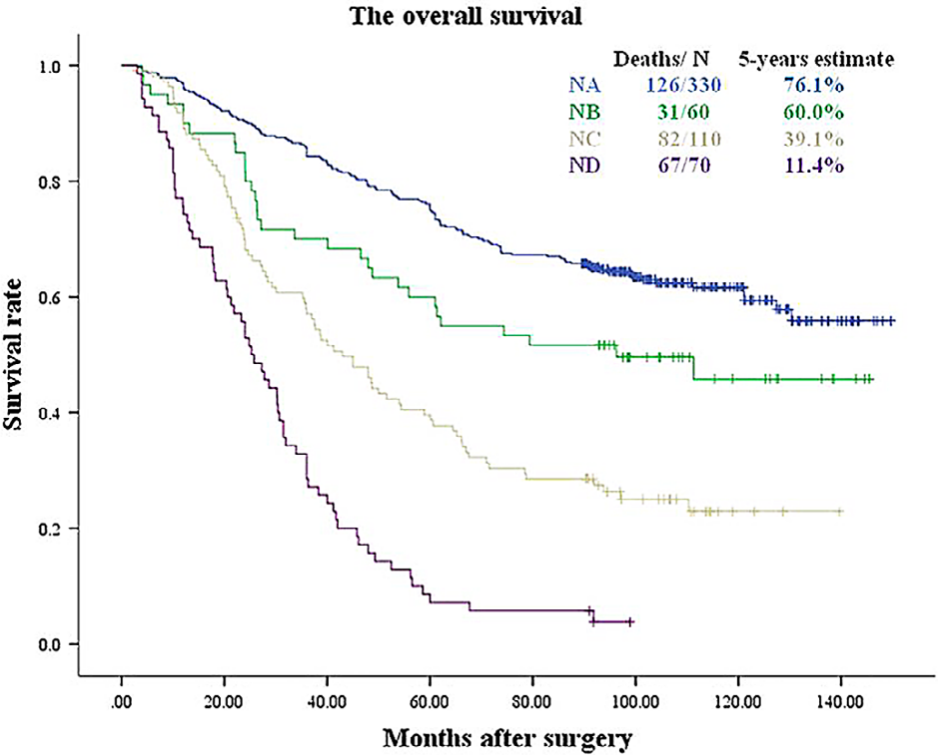
Survival curves for NA, NB, NC and ND of N‐nLNS (Log Rank *P* < 0.001). The differences of survival between neighboring groups were statistically significant (*P*‐values: between NA and NB, *P* = 0.023; between NB and NC, *P* = 0.003; between NC and ND, *P* < 0.001).

When categorized based on nLNS, 330 cases (57.9%) were NA, 60 (10.5%) were NB, 110 (19.3%) were NC, and 70 (12.3%) were ND. Figure 6 shows that there were statistically significant differences among the nodal groups. The five‐year survival rates were 76.1%, 60.0%, 39.1% and 11.4% for NA, NB, NC and ND, respectively (between NA and NB, *P* = 0.023; between NB and NC, *P* = 0.003; between NC and ND, *P* < 0.001). Table 5 shows that the N‐nLNS and pT categories were independent risk factors.

**Table 5:** Results of univariate and multivariate analyses of N‐nLNS (Cox regression model)

	Univariate analyses			Multivariate analyses		
	HR	95%CI	*P*‐value	HR	95%CI	*P*‐value
N‐nLNS			<0.001			<0.001
NA versus NB	0.635	0.429–0.941	0.024	0.665	0.447–0.990	0.044
NB versus NC	0.516	0.341–0.781	0.002	0.534	0.352–0.811	0.003
NC versus ND	0.437	0.315–0.606	<0.001	0.464	0.334–0.646	<0.001
Age						
<60 years versus ≥60 years	0.896	0.715–1.124	0.344			
Sex						
Male versus Female	1.161	0.918–1.467	0.212			
Smoking history						
Yes versus No	1.221	0.974–1.531	0.084			
Location of tumor						
Right lung versus left lung	0.850	0.680–1.064	0.157			
Pathological type			0.526			
Adenocarcinoma versus squamous cell carcinoma	0.988	0.780–1.251	0.920			
Adenocarcinoma versus others	0.775	0.497–1.209	0.261			
Squamous cell carcinoma versus others	0.785	0.498–1.237	0.297			
pT categories			<0.001			<0.001
pT1 versus pT2	0.566	0.432–0.742	<0.001	0.688	0.523–0.906	0.008
pT2 versus pT3	0.653	0.480–0.889	0.007	0.712	0.522–0.971	0.032
pT3 versus pT4	0.602	0.383–0.946	0.028	0.551	0.348–0.870	0.011

## Discussion

Given the continuous development of diagnostic and treatment strategies, the current N classification for lung cancer is unsatisfactory for clinical needs. This is especially important in some N1 or N2 cases, and in these groups, it is necessary to further subdivide patients into subgroups with different prognoses.^12^ Among other organ malignancies, the N staging in these groups may be influenced by different parameters. Similar to gastric cancer, an N classification is determined by the quantity of metastatic lymph nodes.^13^ Therefore, because there are differences among the prognoses of patients with NSCLC, it is imperative to revise the N classification. Therefore, some scholars have attempted to more deeply study the relationships among other conditions related to lymph node involvement and prognosis more in lung cancer. Recently, several articles have studied the effect of the number of involved lymph nodes (nN) on prognosis in patients with lung cancer. Wei *et al*. divided lung cancer patients into four groups according to nN: the absence of metastatic lymph nodes was defined as nN0, 1 to 2 metastatic lymph nodes was defined as nN1, 3 to 6 metastatic lymph nodes was defined as nN2, and seven or more metastatic lymph nodes was defined as nN3. The results of their study showed that as nN increased, the five‐year overall survival of patients significantly decreased. In addition, nN was superior to the anatomical location of metastatic lymph nodes as a prognostic determinant.^14^ Other studies have also confirmed that the amount of lymph node involvement is an independent prognostic factor associated with survival in patients undergoing surgery for NSCLC.^15^, ^16^ Saji *et al*. presented a new proposal to combine N and nN (N‐nN) as a new classification for lymph nodes. They divided the N1 or N2 cases into two groups according to whether the nN was higher or lower than three. Their results revealed that N‐nN was more accurate than the current N classification as a prognostic factor, especially in some prognostically heterogeneous N1 or N2 patients with NSCLC.^17^

In addition, the proportion of positive lymph nodes out of the total number of resected lymph nodes (i.e., the ratio of involved lymph nodes, LNR) has also been explored in some studies. Based on the LNR, the study of Nwogu *et al*. classified patients into three levels: low LNR (0.01% to 24%), moderate LNR (25% to 49%), and high LNR (50% and above). Prognoses were better in patients with a low or moderate LNR than in those with a high LNR.^18^ Taylor *et al*. also came to a similar conclusion in their study, in which they found that in patients undergoing complete resection for NSCLC, a higher LNR resulted in lower survival rates and a shorter time to recurrence after surgery.^19^ Ding *et al*. comprehensively compared five lymph node classifications, including N, nN, N‐nN, LNR, and a combination consisting of N and LNR (N‐LNR). They concluded that while all five of these methods were prognostic for patients with NSCLC, N‐LNR was the best predictor of survival.^20^

Compared with the lymph node classifications mentioned above, the classification proposed in the present study is more related to the ideas presented by the IASLC, which suggests considering the impact of N and nLNS on survival. On the one hand, nLNS was found to be a better prognosis predictor. In on study (Kang *et al*.), N, nN, and nLNS were prognostic factors associated with overall survival in univariate analyses. However, only nLNS was an independent risk factor that affected prognoses in multivariate analyses.^21^ Similarly, Riquet *et al*. also found that overall survival was related to nLNS and not to nN.^22^ On the other hand, the amount of lymph nodes harvested varies substantially from one patient to another.^22^, ^23^ Our study similarly found that there was a large amount of fluctuation in the number of harvested lymph nodes (1 to 12) and positive lymph nodes (0 to 9) among stations. It is difficult to precisely count the number of lymph nodes.^20^ It is possible that using nN and LNR to assess a prognosis causes the abovementioned problems to a certain degree. Implementing the use of nLNS could avoid these problems.

In this study, 570 patients were first divided into N0, N1, and N2 groups according to N. Then, based on the number of involved lymph node stations in each region (single vs. multiple), N1 was further divided into N1a and N1b, and N2 was divided into N2a and N2b. Our results show that a prognosis of N1a is better than a prognosis of N1b and that N2a is better than N2b. Using the IASLC also provided the same results.^8^, ^9^ Fujimoto *et al*. reported that in patients with N1 NSCLC, the recurrence‐free survival rate was related to nLNS, and the rates of tumor recurrence and distant metastasis were higher in N1b than in N1a.^24^ However, other authors have reached different conclusions. For example, Asamura *et al*. found that there was no significant difference in survival between N1a and N1b, while N1a showed a trend toward better survival and had a higher five‐year survival rate (73%) than was found for N1b (54%).^25^ Regarding the effect of nLNS on the prognosis of N2 cases, two Japanese studies provided support for our view that the survival rate is significantly better for N2a than for N2b disease.^26^, ^27^ However, Luzzi *et al*. presented a different findings and reported that there was no significant difference between N2a and N2b.^28^

The five‐year survival rate was better in N2 patients with skip metastasis than in those with no skip metastasis.^29^ By considering the presence or absence of skip metastasis (i.e., with or without N1 lymph node metastasis), the IASLC further classified N2a into N2a1 and N2a2. The survival rate was significantly better in N2a1 than in N2a2.^8^ In our study, N2b was divided into N2b1 and N2b2 based on the same requirements. Our results showed that the survival curves for N2a1 and N2a2 overlapped significantly with each other and that there was no significant difference in survival between these two groups. We assumed that the number of N2a cases was very small, and this was why the result achieved in this group was not similar to those of the IASLC. However, there was significant difference in survival between N2b1 and N2b2. Therefore, N2a was no longer subdivided, and the subgroups within N2b were retained. In the next step, we pairwise compared overall survival among N0, N1a, N1b, N2a, N2b1, and N2b2. The prognoses for N0, N1a and N2b2 were significantly different from those of the other five groups. However, there were no differences in survival among N1b, N2a and N2b1. In an article about the seventh edition N classification, the IASLC divided N1 and N2 categories into N1a, N1b, N2a and N2b according to the number of involved nodal zones. The survival curves for N1b and N2a were superimposed, and the IASLC therefore merged N1b and N2a into one prognostic group. Finally, the N1 and N2 categories were classified into three distinct prognostic groups: single‐zone N1, multiple‐zone N1/single‐zone N2, and multiple‐zone N2.^9^ In an article about the eighth edition N classification, the IASLC reported that the survival curves for N1b intersected with the curves for N2a2, and the prognosis of N2a1 was slightly better than that of N1b, although the difference was not significant.^8^ Other articles have also reached similar conclusions. Asamura *et al*. reported that survival was better in some N1 cases survival than in N2b; however, the survival curves for N1 overlapped with N2a.^25^ Keller *et al*. found that in patients with left upper lobe NSCLC, the survival rate was similar between N2a and N1.^30^ The general rules of the TNM classification for malignant tumors states that groups with similar prognoses can be combined.^31^ Hence, some N1 and N2 cases with similar prognoses (e.g., N1b, N2a and N2b1) were combined into one group, and the other three groups (N0, N1a and N2b2), among which the prognosis was significantly different, were left as they were. Eventually, for the exploratory analysis performed for our study, nodal disease was coded as NA (i.e., N0), NB (i.e., N1a), NC (i.e., N1b, N2a and N2b1) and ND (i.e., N2b2) according to N‐nLNS. Further Kaplan‐Meier and Cox regression analyses confirmed that N‐nLNS effectively predicted postoperative survival in patients with lung cancer.

Our research was inspired by the study of Asamura *et al*.^8^ The main purpose of our study was to validate the feasibility of their hypothesis regarding different N classifications (N‐nLNS) among our patients. However, there are two important differences between our and their studies. On the one hand, in our study, N2b was further divided into N2b1 and N2b2, and we found that there was a significant difference in survival between these two groups. Our study also showed that skip metastasis played a role in N2b patients. On the other hand, because of their similar prognoses, some groups (i.e., N0, N1a, N1b, N2a1, N2a2, N2b1 and N2b2) were merged on the basis of N‐nLNS into new groups (i.e., NA, NB, NC and ND).

Although our study led us to conclude that N‐nLNS is a more sophisticated method for assessing the tumor burden in lymph nodes, we further allocated lung cancer patients to more accurate prognostic groups. In the future, this may help to provide more personalized postoperative adjuvant therapy in affected patients. However, there are some limitations to our study. First, this was a retrospective study, and the number of included cases was low. A different group of patients (e.g., patients with different races, regions, pathological types, etc) might yield different results. Although N‐nLNS was shown to be feasible in our patients, this may not universally true. In addition, there are too many subgroups in NC. Our study included too few NC cases to further study the true relationships among N1b, N2a and N2b1. However, we were able to gain some insight and found that N categories were no longer determined based only on the location of the involved lymph nodes and that combining N with nLNS may be a better choice in the future. Therefore, a prospective trial with a large sample size will be required for a more in‐depth study and could even be used to perform sufficient subgroup analyses, such as IASLC, to verify the feasibility of N‐nLNS, which should be constantly revised. Second, this classification of lymph nodes is based on the pathological findings obtained in postoperative lymph node specimens and cannot be effectively used in clinical settings. Which lymph nodes are involved preoperatively or in non‐surgical patients can be determined based on an imaging examination or biopsy. The study of Silvestri GA *et al*. reported that a preoperative examination was useful in identifying mediastinal lymph node metastasis and found that the sensitivity and specificity of CT were approximately 55% and 81%, respectively, while those of PET were approximately 77% and 86%, respectively. Minimally invasive needle techniques had sensitivities of approximately 89% or higher.^32^ Although these common methods can identify some involved lymph nodes, it is difficult to discriminate which lymph node represents metastasis. Therefore, for patients without surgical lymphadenectomy, the current N classification is the best and easiest method to determine their clinical N categories. In spite of this point, N‐nLNS predicted the prognosis of patients after surgery to a certain extent and was better at selecting the appropriate postoperative adjuvant treatment for different patients, especially in some N2 cases with skip metastasis, because their postoperative survival rates were similar to those in some N1 cases. Therefore, the importance of nLNS in pathological N categories cannot be ignored. In clinical practice, breast cancer has two different N classifications, with the clinical N classification determined by an assessment of the anatomical location of the involved lymph nodes and the pathological N classification mainly defined in terms of the number of involved lymph nodes.^33^ Thus, it would be worthwhile to further study whether NSCLC has a revised pathological N classification that is different from that of a clinical N classification, such as breast cancer.

In conclusion, the results of this study demonstrate that N‐nLNS predicts the prognosis of NSCLC patients with involvement of different lymph node stations. In particular, in some N1 or N2 cases, this classification provided advantages over the use of the current N categories. According to this study, some patients with a relatively better prognosis (e.g., N2 cases with skip metastasis) could be distinguished from N2 cases with a relatively poor prognosis and given more active and effective postoperative treatment. The idea of merging the current N classification with other conditions related to lymph node involvement is practical and necessary for present clinical needs. However, before a new N classification can be applied in a clinical setting, a prospective and international database with a large number of cases must be developed so that many further analyses can be performed with different subgroups.

## Disclosure

The author has no conflict of interest,
